# Promoting effect of hepatitis B virus on the expressoin of phospholipase A2 group IIA

**DOI:** 10.1186/s12944-016-0400-7

**Published:** 2017-01-11

**Authors:** Chengliang Zhu, Hui Song, Bingzheng Shen, Long Wu, Fang Liu, Xinghui Liu

**Affiliations:** 1 Department of Clinical Laboratory, Renmin Hospital of Wuhan University, Wuhan, Hubei 430060 People’s Republic of China; 2 Department of Clinical Laboratory, Shanghai Gongli Hospital, the Second Military Medical University, Pudong New Area, Shanghai, 200135 People’s Republic of China; 3 The State Key Laboratory of Virology, College of Life Sciences, Wuhan University, Wuhan, Hubei 430072 People’s Republic of China

**Keywords:** Hepatitis B virus, Phospholipase A2 group IIA, Chronic hepatitis B, Liver cirrhosis, Hepatocellular carcinoma

## Abstract

**Background:**

Hepatitis B virus (HBV) infection causes acute and chronic liver disease, ultimately leading to the development of liver cirrhosis (LC) and hepatocellular carcinoma (HCC). Phospholipase A2 group IIA (PLA2G2A) plays important roles in the development and progression of many tumors. Thus far, there have been no reports on the association between HBV and PLA2G2A. The present study investigated the effect of HBV infection on PLA2G2A expression and its application in the diagnosis of HBV-related diseases.

**Methods:**

Serum levels of PLA2G2A in 308 HBV-infected patients and 185 healthy controls were measured using an enzyme-linked immunosorbent assay (ELISA). The difference in serum levels of PLA2G2A was analyzed among chronic hepatitis B (CHB), LC, and HCC patients. PLA2G2A mRNA and protein expression in HepG2 and HepG2.2.15 cells carrying the integrated HBV genome were measured using reverse transcription polymerase chain reaction (RT-PCR) and western blot assays. The HBV infectious clone pHBV1.3, the control plasmid pBlue-ks and PLA2G2A gene promoter were transfected into HepG2 and HepG2.2.15 cells. After transfection, the luciferase activity was measured in the cells. PLA2G2A mRNA and protein expression levels were examined using RT-PCR and western blot assays.

**Results:**

The serum levels of PLA2G2A were 258.3 ± 20.3ng/dl in the healthy controls and 329.0 ± 22.5ng/dl, 385.4 ± 29.3ng/dl and 459.2 ± 38.6ng/dl in the CHB, LC, and HCC patients, respectively. Statistical analyses revealed significantly higher serum levels of PLA2G2A in CHB, LC, and HCC patients than in the healthy controls (*P* < 0.05), and PLA2G2A levels were elevated in the order of HCC > LC > CHB group. High serum PLA2G2A levels in HCC patients were associated with a lower prevalence of lymph node metastasis and a lower TNM stage. HepG2.2.15 cells carrying the HBV genome expressed higher levels of PLA2G2A mRNA and protein than the HepG2 cells. In addition, HBV triggered PLA2G2A promoter activity and enhanced PLA2G2A mRNA and protein expression compared to the empty vector pBlue-ks.

**Conclusion:**

HBV can upregulate the expression of PLA2G2A, and serum levels of PLA2G2A are associated with the progression of HBV-related diseases.

## Background

Hepatocellular carcinoma (HCC) is the third leading cause of cancer-related deaths. Hepatitis B virus (HBV) infection is a major factor for HCC development. Chronic hepatitis B (CHB) may lead to the development of liver cirrhosis (LC) and HCC. It is estimated that the risk of developing HCC is 200 times higher in chronic HBV-infected patients than in the general population without HBV infection [1–4]. However, the carcinogenic mechanism of HBV-related HCC is still poorly understood. It is generally considered that HBV infection is noncytopathic [5, 6]. Instead, considerable evidence has shown an immune and inflammatory contribution to liver dysfunction, HBV infection activates a number of cellular genes including interleukin 27 (IL-27), IL-29, IL-8 and cyclooxygenase 2 [7–9]. In a previous research, we screened differentially expressed genes in HepG2.2.15 cells and HepG2 cells using gene chips [10]. Phospholipase A2 group IIA (PLA2G2A) was identified as a highly expressed gene in HepG2.2.15 cells (data not shown).

PLA2G2A is a secreted protein that is a member of the phospholipase A2 family. PLA2G2A is widely present in various mammalian tissues, such as the lung, thymus, liver, kidney, and prostate [11, 12]. PLA2G2A is closely associated with the inflammatory and immune response in the body [12, 13], and it also plays an important role in the development and progression of tumors [14, 15]. The aim of the present study was to investigate the effect of HBV infection on PLA2G2A expression, its application in the diagnosis of HBV-related diseases, and the underlying molecular mechanism. The results will provide new insights as to the pathogenesis of HBV and for the diagnosis of HBV-related diseases.

## Methods

### Study subjects

In total, 308 patients clinically diagnosed with chronic HBV infection were recruited. According to the clinical, biochemical, serological, histopathological, abdominal ultrasound, computed tomography (CT) and magnetic resonance imaging (MRI) examination results, the patients were divided into three groups: 143 CHB patients, including 81 men and 62 women, with a mean age of 43.2 ± 16.7 years, 86 LC patients, including 48 men and 38 women, with a mean age of 50.5 ± 18.5 years,and 79 HCC patients, including 49 men and 30 women, with a mean age of 59.6 ± 17.3 years. All patients with diseases affecting the heart, brain, and kidneys (among various other vital organs) and those with other hepatotropic virus infections were excluded. The control group included 185 healthy examinees, including 105 men and 80 women, with a mean age of 48.6 ± 20.1 years.

### Cell culture and transfection

HepG2 and HepG2.2.15 cells carrying the integrated HBV genome [16] were cultured in RPMI-1640 medium supplemented with 10% fetal bovine serum. The cultures were incubated in a cell incubator at 5% CO_2_ and 37°C. HepG2 cells were seeded into 6- or 24-well cell plates before transfection. When the cells reached approximately 80% confluency, 2 μg of plasmid DNA and 2 μL of Lipofectamine 2000 (Invitrogen, U.S.A) were diluted in 30 μL of Dulbecco’s Modified Eagle Medium (DMEM), or 4 μg of plasmid DNA and 6 μL of Lipofectamine 2000 reagent were diluted in 100 μL of RPMI-1640. The reactions were allowed to proceed at room temperature for 20 min. The prepared transfection solution was added to the 24- or 6-well cell plates, and the cells were further incubated in a CO_2_ incubator.

### Reverse transcription polymerase chain reaction (RT-PCR) assay

Total RNA was extracted from HepG2 and HepG2.2.15 cells using TRIzol reagent (Invitrogen, Carlsbad, CA, USA) [17]. The cDNA was synthesized using M-MLV reverse transcription. PCR amplification for PLA2G2A was verified using the following primers:

- PLA2G2A sense: 5′ GCACTCAGTTATGGCTTCT3′ and
- PLA2G2A anti-sense: 5′ ATTGTAGGTCGTCTTGTTTC 3′.
- β-actin was amplified as a control. The PCR products were checked using 1% agarose gel electrophoresis.

### Luciferase assay

After transfection, HepG2 and HepG2.2.15 cells were cultured for an additional 48 h. The cell supernatants were removed, and the cells were harvested for lysis with a cell lysis buffer. After lysis, 10 μL of cell lysate was mixed with 100 μL of luciferase substrate, and the optical density was measured using a luminometer. Each sample was tested in triplicate.

### Western blot assay

HepG2 cells were harvested and lysed, and then 30 μg of protein from each sample was mixed with an equal volume of 5X loading buffer, which was then boiled at 100°C for 5 min and separated using 12% SDS-PAGE gel electrophoresis. The proteins were then transferred to a nitrocellulose membrane and blocked with 5% skim milk for 2 h. The membrane was incubated with the PLA2G2A monoclonal antibody (1:1000) for 2 h. The membrane was washed with PBST three times and then incubated with anti-rabbit secondary antibody (Sigma, 1:5000) for 1 h. After four washes with PBST, the membrane was subjected to color development using an electrochemiluminescence (ECL) detection system (Amersham Life Sciences).

### Enzyme-linked immunosorbent assay (ELISA)

Approximately 2 mL of fasting venous blood was collected from each subject. Serum levels of PLA2G2A were measured using an ELISA kit (Cayman Chemical, AnnArbour, MI, USA) following the manufacturer’s instructions. Each sample was tested in triplicate.

### Statistical analysis

Statistical analyses were performed using the Statistical Package for the Social Sciences (SPSS) 16.0 statistical package. The data are expressed as the mean ± standard deviation $\left(\overline{\mathrm{x}}\kern0.5em \pm \kern0.5em \mathrm{s}\right)$, and a logistic regression analysis was performed adjusted for age. The differences among the healthy controls and the patients with CHB, LC and HCC were assessed using one-way ANOVA, and a two tailed P-value <0.05 was considered statistically significant.

## Results

### Subjects

The demographic and clinical characteristics of the subjects are shown in Table 1. There were no significant differences in gender and body mass index (BMI) among the 4 groups (*P* > 0.05). The subjects with more progressive disease tended to be older. The aspartate transaminase (AST) and alanine transaminase (ALT) levels were higher in the CHB, LC, and HCC patients compared with the healthy controls (*p* < 0.05), no significant difference existed among the CHB, LC, and HCC patients in terms of the HBV DNA (*P* > 0.05).

**Table 1:** Baseline characteristics of the subjects enrolled in the study

Characteristic	Healthy controls (*n* = 185)	CHB patients (*n* = 143)	LC patients (*n* = 86)	HCC patients (*n* = 79)
Age (years)	48.6 ± 20.1	43.2 ± 16.7	50.5 ± 18.5	59.6 ± 17.3
Gender (M/F)	105/80	81/62	48/38	49/30
BMI(kg/m^2^)	26.3 ± 1.7	24.6 ± 1.5	25.2 ± 1.8	24.2 ± 1.6
ALT(IU/l)	<30	183.5 ± 105.7	126.5 ± 98.4	65.7 ± 43.2
AST(IU/l)	<30	207.4 ± 128.6	113.4 ± 86.5	83.2 ± 56.8
HBV DNA (Lg copies/ml)	0	5.7 ± 2.6	5.5 ± 2.2	5.3 ± 2.4

*Abbreviations*: *n* number of the subjects, *NS* none sense, *M* male, *F* female, *BMI* body mass index, *ALT* alkanine aminotransferase, *AST* aspartate aminotransferase

### Serum levels of PLA2G2A are elevated in HBV patients

**Fig. 1:**
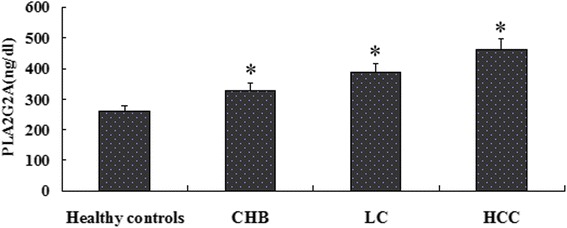
Serum PLA2G2A levels in healthy controls and HBV patients. The serum PLA2G2A levels in the healthy controls and in CHB, LC, and HCC patients were measured using an ELISA. *P < 0.05

Secreted PLA circulates in the blood stream and in virtually every tissue in mammals. We then measured the serum levels of PLA2G2A in healthy controls and in CHB, LC, and HCC patients using an ELISA. The results showed that the serum levels of PLA2G2A were 258.3 ± 20.3ng/dl, 329.0 ± 22.5ng/dl, 385.4 ± 29.3ng/dl, and 459.2 ± 38.6ng/dl in the healthy controls, CHB patients, LC patients, and HCC patients, respectively. In the logistic regression analyses adjusted by age, we found that that compared with the healthy controls, the HBV patients had significantly higher serum levels of PLA2G2A (*P* < 0.05), Furthermore, among the various groups of patients, the serum levels of PLA2G2A consistently increased with the progression of HBV diseases which is in the order of HCC > LC > CHB patients (Fig. 1).

We further analyzed the relationship between the serum levels of PLA2G2A and the clinical characteristics in HCC patients. The results indicated that no significant associations were found between serum PLA2G2A levels and clinical factors including age and gender distribution (*P* > 0.05), whereas higher serum PLA2G2A levels were associated with less frequent lymph node metastasis and lower TNM stages (Table 2).

**Table 2:** Association between PLA2G2A expression and clinical characteristics in HCC patients

Characteristics	Number	Serum PLA2G2A levels(ng/dl)	*P* value
Gender			
Male	49	460.6 ± 40.3	0.472
Female	30	456.7 ± 36.5	
Age (year)			
≥ 60	53	458.5 ± 37.4	0.424
< 60	26	462.2 ± 41.7	
Lymph node metastasis			
Negative	47	482.6 ± 53.8	0.028
Positive	32	422.6 ± 31.3	
TNM stages			
I + II	28	491.7 ± 55.3	0.018
III + IV	51	415.8 ± 31.5	

*Abbreviations*: *n* number of the subjects, *TNM* tumour node metastasis, *PLA2G2A* phospholipase A2 group IIA

### HBV increases PLA2G2A mRNA and protein expression

**Fig. 2:**
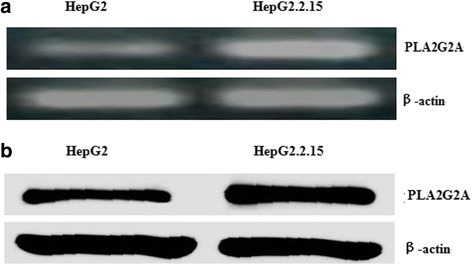
PLA2G2A mRNA and protein expression in HepG2 and HepG2.2.15 cells. **a** The relative mRNA levels of PLA2G2A in the HepG2 and HepG2.2.15 cells were measured using RT-PCR analysis. **b** PLA2G2A protein expression in HepG2 and HepG2.2.15 cells was measured using western blotting

HepG2.2.15 cells were stably transfected with the complete HBV genome, which expressed HBV RNA and viral proteins and produced virus-like particles [18]. To assess the effect of HBV on PLA2G2A expression, we analyzed PLA2G2A mRNA and protein expression in HepG2 and HepG2.2.15 cells using RT-PCR and western blot assays. The results showed that HepG2.2.15 cells expressed significantly higher levels of PLA2G2A mRNA and protein than the HepG2 cells (Fig. 2a and b).

### PLA2G2A gene promoter activity is triggered by pHBV1.3

**Fig. 3:**
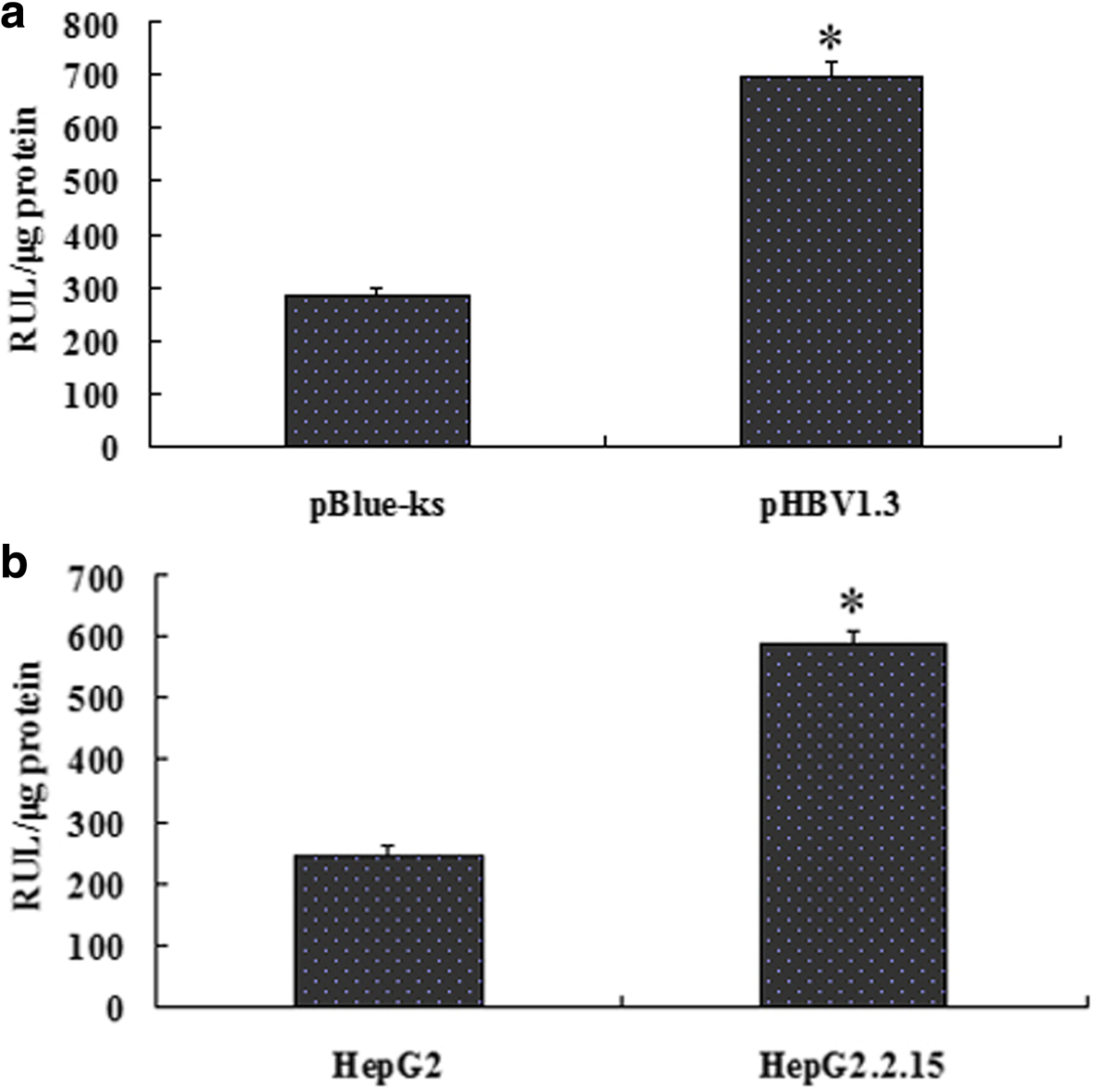
Effect of HBV on the activity of the PLA2G2A promoter. **a** HepG2 cells were co-transfected with pHBV1.3/pBlue-ks and the PLA2G2A promoter pPLA2G2A-Luc plasmid, and then luciferase activity was measured. **b** HepG2 and HepG2.2.15 cells were transfected with PLA2G2A promoter pPLA2G2A-Luc plasmid, and then luciferase activity was measured. *P < 0.05

PHBV1.3 is an infectious clone of HBV. After transfection with pHBV1.3, HepG2 cells can synthesize and secrete HBV viral particles [9]. To investigate the molecular mechanism by which HBV regulates PLA2G2A expression, we co-transfected the HBV infectious clone pHBV1.3 and the PLA2G2A gene promoter pPLA2G2A-Luc into HepG2 cells, and pBlue-ks was transfected as a control. Additionally, pPLA2G2A-Luc was transfected into HepG2 and HepG2.2.15 cells respectively. The results of a luciferase activity assay showed that the PLA2G2A gene promoter activity was significantly enhanced in the HepG2 cells after transfection with pHBV1.3 (692.5 ± 28.8 RUL/μg protein, *P* < 0.05) compared with the control (279.6 ± 16.7 RUL/μg protein), and luciferase activity was much higher in HepG2.2.15 cells (588.1 ± 21.3 RUL/μg protein, *P* < 0.05) than in the HepG2 cells (243.2 ± 15.5 RUL/μg protein). This result indicated that HBV triggered PLA2G2A gene promoter activity (Fig. 3a and b).

### PHBV1.3 increases the PLA2G2A mRNA and protein expression

**Fig. 4:**
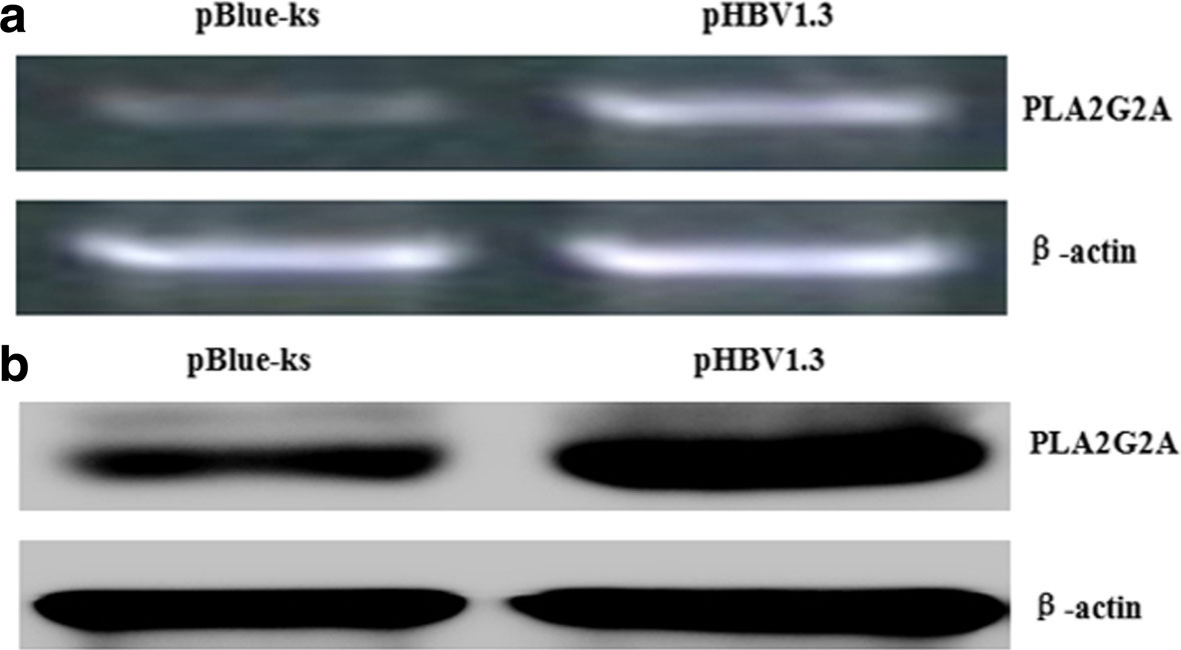
Effect of pHBV1.3 on PLA2G2A mRNA and protein expression. **a** Effects of pHBV1.3 on the expression of PLA2G2A mRNA. HepG2 cells were transfected with pHBV1.3 or pBlue-ks, and then 48 h after transfection, PLA2G2A mRNA was measured by RT-PCR analysis. **b** Effects of pHBV1.3 on the expression of PLA2G2A protein. HepG2 cells were transfected with pHBV1.3 or pBlue-ks, and then 48 h after transfection, PLA2G2A protein was measured using western blotting

We transfected pHBV1.3 into HepG2 cells and used an empty vector transfection with pBlue-ks as a control. Then, we analyzed the PLA2G2A mRNA and protein expression using RT-PCR and western blot assays, respectively. The results showed that compared with the control, the PLA2G2A mRNA and protein expression levels were increased in the HepG2 cells after transfection with pHBV1.3 (Fig. 4a and b).

## Discussion

HBV is currently recognized as one of the main causes of HCC. The mechanism by which HBV infection leads to HCC is complex, involving both the host and viral factors [19]. For example, HBV can inactivate the tumor suppressor gene P53 in the body, leading to uncontrolled cell proliferation and endangering critical biological functions, such as DNA repair and control [20]. Additionally, HBV viral infection can induce a strong T cell immune response in the host. However, this immune response kills hepatocytes, which leads to inflammation and causes the regeneration of many hepatocytes, inducing a carcinogenic effect [21]. Moreover, HBV can regulate the expression of particular genes in the host, however, these genes participate in the development and progression of HCC [7]. Our previous research showed that HBV can increase the expression of collagen triple helix repeat containing-1(CTHRC1), which is a protein that plays a major role in promoting hepatocyte proliferation, migration, and invasion [22, 23].

In the present study, we found that HepG2.2.15 cells expressed significantly higher levels of PLA2G2A mRNA and protein than HepG2 cells. The serum levels of PLA2G2A in HBV patients were significantly elevated and associated with disease progression, lymph node metastasis and TNM stage. We further demonstrated that HBV increases PLA2G2A mRNA and protein expression by triggering the activity of its gene promoter.

HepG2.2.15 cells have a HBV stably integrated into its genome and are capable of producing HBV-like viruses. The cells are mostly arrested in the G1 phase. There is a reduction of filopodia, actin and ezrin in HepG2.2.15, which makes them less invasive after implantation in nude mice than the HepG2 parental line, and the HepG2.2.15 implanted cells caused liver necrosis, fatty liver, high cholesterol, degenerative changes, and neutrophil infiltration [24]. This study found that HepG2.2.15 cells had significantly increased PLA2G2A levels compared with HepG2 cells.

PLA2G2A expression was elevated in primary gastric, colon, and prostrate tumors and β-catenin–dependent Wnt signaling is a major upstream regulator of PLA2G2A expression [25]. Hepatitis B virus X (HBX) protein upregulates β-catenin and the Wnt/β-catenin pathway is frequently activated in HBV-induced HCC [19, 26], which suggests that HBV might upregulate the expression of PLA2G2A via the Wnt signaling pathways.

Recent studies showed that PLA2G2A is closely associated with the development and progression of particular tumors [15, 27–29]. PLA2G2A can stimulate tumor cell growth, whereas the product of PLA2G2A, arachidonic acid, leads to prostate tumor cell proliferation and facilitates tumor angiogenesis and metastasis. However, PLA2G2A expression was decreased in metastatic and late-stage tumors and is associated with prolonged survival and less frequent metastasis in gastricadenocarcinoma [25]. In the present study, we found that elevated PLA2G2A expression was associated with less frequent lymph node metastasis and lower TNM stages. However, this study has certain limitations such as the small sample size in the patient subgroups, and the evaluation of the correlation of serum PLA2G2A levels with the prognosis of HCC patients needs to be evaluated. In addition, the detailed molecular mechanism of how HBV induces the promoter activity of PLA2G2A needs further investigation.

## Conclusions

Taken together, for the first time, we demonstrated the dynamic changes in PLA2G2A expression in the progression of HBV infection ranging from CHB, LC to HCC. Therefore, measuring the serum levels of PLA2G2A in HBV patients may provide a new biomarker for the diagnosis of progressive liver diseases during chronic HBV infection.
